# Research Progress on Glioma Microenvironment and Invasiveness Utilizing Advanced Multi-Parametric Quantitative MRI

**DOI:** 10.3390/cancers17010074

**Published:** 2024-12-29

**Authors:** Dandan Song, Guoguang Fan, Miao Chang

**Affiliations:** Department of Radiology, The First Hospital of China Medical University, 155 Nanjing North Street, Heping District, Shenyang 110001, China; liuchang8600@sina.com

**Keywords:** glioma, multi-parametric quantitative MRI, tumor microenvironment, invasive, genotyping

## Abstract

Magnetic resonance imaging (MRI) currently serves as the primary diagnostic method for glioma detection and monitoring. The integration of neurosurgery, radiation therapy, pathology, and radiology in a multi-disciplinary approach has significantly advanced its diagnosis and treatment. However, the prognosis remains unfavorable due to treatment resistance, inconsistent response rates, and high recurrence rates after surgery. These factors are closely associated with the complex molecular characteristics of the tumors, the internal heterogeneity, and the relevant external microenvironment. The complete removal of gliomas presents challenges due to their infiltrative growth pattern along the white matter fibers and perivascular space. Therefore, it is crucial to comprehensively understand the molecular features of gliomas and analyze the internal tumor heterogeneity in order to accurately characterize and quantify the tumor invasion range. The multi-parameter quantitative MRI technique provides an opportunity to investigate the microenvironment and aggressiveness of glioma tumors at the cellular, blood perfusion, and cerebrovascular response levels. Therefore, this review examines the current applications of advanced multi-parameter quantitative MRI in glioma research and explores the prospects for future development.

## Simple Summary

Due to the complex heterogeneity, inherent high invasiveness, and high recurrence rate of gliomas, the prognosis for patients is extremely unfavorable, especially in the case of glioblastoma. Extensive tumor resection can enhance patient prognosis; however, the diffuse infiltrative growth and malignant characteristics of glioma pose challenges in achieving complete tumor resection. Therefore, we present a review of advanced multi-parameter quantitative MRI applications at the biological level in terms of the cellular, blood perfusion, and cerebrovascular response to explore its feasibility in assessing the complex intra-tumoral heterogeneity and visualizing the extent of peri-tumoral invasion, while providing guidance for future research and clinical implementation.

## 1. Introduction

Glioma, accounting for 26.3% of all central nervous system (CNS) tumors [1], is the predominant malignant primary brain tumor in adults and ranks as the second leading cause of death from intracranial primary diseases following stroke [2]. The annual incidence rate in the population ranges from 47% to 57% [3], whereas the overall survival duration is approximately 12–15 months [4]. The most aggressive form of brain tumor, glioblastoma (GBM), exhibits the highest incidence rate among them, accounting for 14.2% of all tumors and 50.9% of all malignant tumors [1]. Despite the standard Stupp treatment regimen [5], which entails aggressive tumor resection followed by chemoradiotherapy and targeted therapy, the overall prognosis remains unfavorable [6], with a significantly low long-term survival rate. The median survival duration is approximately 15 months, and less than 5% of patients survive beyond 5 years [3]. Furthermore, as their neurological function and quality of life gradually deteriorate, the associated complications can have a profound impact on both patients and their families. The emergence of precision oncology and immunotherapy has led to the development of more efficacious and well-tolerated treatments for this malignant and aggressive disease [7].

The 2021 World Health Organization (WHO) classification of tumors of the CNS, CNS 5th edition [8], has been updated to incorporate molecular and histopathological findings based on the 2016 CNS 4th edition [9]. The classification of diffuse gliomas in adults is now as follows [10]: (1) astrocytoma—isocitrate dehydrogenase (IDH) mutant, WHO grade 2–4; (2) oligodendroglioma—IDH mutant without 1p/19q co-deletion, WHO grade 2–3; (3) GBM—IDH wildtype, WHO grade 4. Notably, IDH mutations are associated with a longer overall survival [8], approximately lasting for about 57 months, which is commonly observed in WHO grade 2–3 gliomas. Telomerase reverse transcriptase (TERT) mutations are associated with a more aggressive disease course, leading to a significantly lower overall survival rate [11,12,13], approximately 11.5 months, particularly in GBM. In addition, the homozygous deletion of CDKN2A/B, which is a tumor suppressor gene located on chromosome 9 and second only to p53 mutation in importance, serves as an indicator of poor prognosis according to the WHO CNS 5th edition. This genetic alteration alone is sufficient to classify a tumor as WHO grade 4, even without the presence of micro-angiogenesis or necrosis. Furthermore, the CDKN2A/B homozygous deletion mutation can also be utilized as a molecular marker for oligodendroglioma classified as WHO grade 3 [8,14]. Relevant studies have reported that approximately 60% of GBM patients present CDKN2A gene deletions, which reshape the cancer cell lipid metabolism and induce ferroptosis in GBM—a novel target for tumor therapy [15].

**Figure 1:**
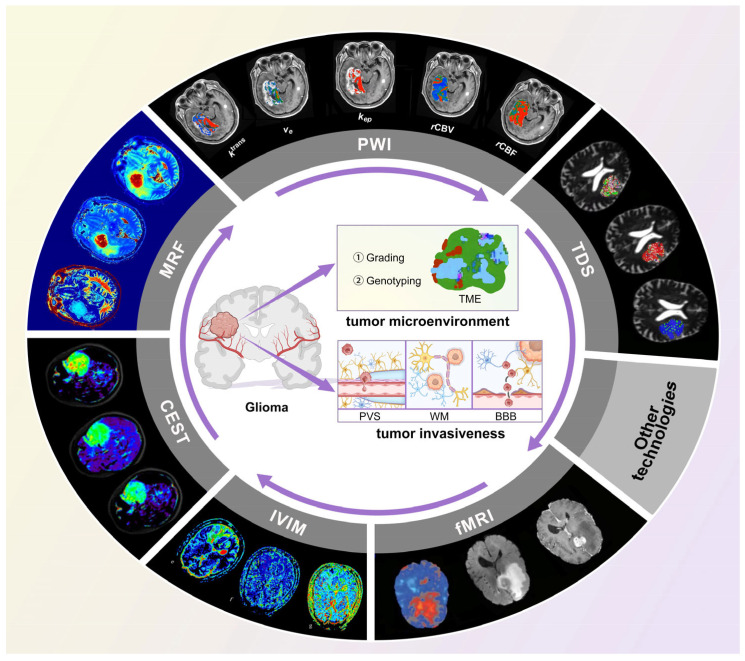
A framework diagram exhibiting the available applications of advanced multi-parametric quantitative MRI in exploring the glioma microenvironment and invasiveness. Abbreviations: MRI, magnetic resonance imaging. PWI, perfusion-weighted imaging. TDS, time-dependent diffusion-weighted imaging. fMRI, functional magnetic resonance imaging (reprinted with permission from Ref. [16]. 2018, Englander ZK et al.). CEST, chemical exchange saturation transfer (reprinted with permission from Ref. [17]. 2018, Paech D et al.). IVIM, intra-voxel incoherent motion (reprinted with permission from Ref. [18]. 2023, Guo, D et al.). MRF, magnetic resonance fingerprinting. TME, tumor microenvironment. PVS, perivascular space. WM, white matter. BBB, blood–brain barrier. rCBV, relative cerebral blood volume. rCBF, relative cerebral blood flow.

Currently, conventional qualitative MRI techniques have limitations in accurately delineating the boundaries of invasive gliomas, distinguishing between tumor and non-tumor components, and providing comprehensive biological information on the intra-tumoral heterogeneity, particularly with regard to post-treatment changes and tumor recurrence. Consequently, advanced neuro-oncology imaging research is focused on addressing these challenges. Extensive investigations are being conducted on magnetic resonance fingerprinting (MRF), intra-voxel incoherent motion (IVIM), multiple diffusion metrics imaging (DXI), temporal diffusion spectroscopy (TDS), functional magnetic resonance imaging (fMRI), magnetic resonance perfusion weighted imaging (PWI), and chemical exchange saturation transfer (CEST), along with other emerging MRI technologies, for their potential applications in tumor diagnosis, grading, and prognosis and the characterization of intra-tumoral heterogeneity, as well as post-treatment evaluation (Figure 1). The objective of this review is to provide a comprehensive overview of the advancements made in these domains, thereby enhancing the precision and efficacy of brain tumor management while offering valuable insights into the current status of advanced MRI technology for glioma and its potential clinical application.

## 2. Glioma Microenvironment

The high aggressiveness and poor prognosis of gliomas, especially GBM, can be attributed to the significant and variable intra- and inter-tumor heterogeneity to some extent [19]. Previous studies have demonstrated that gliomas are not simple homogeneous entities but rather a collection of multiple heterogeneous subregions [20]. The spatial heterogeneity observed in tumors may stem from the uneven distribution of tumor cell subpopulations harboring distinct genetic mutations throughout the tumor or from the prevalence of clonal populations of tumor stem cells that establish differentiation gradients and occupy specific subregions [21,22]. The identification of genotypes, although highly significant, remains insufficient in fully characterizing the dynamic changes occurring in the entire tumor [23]. Over time, tumor evolution is typically influenced by complex factors within the tumor microenvironment (TME), such as local blood perfusion, the oxygen concentration, metabolism, and the immune response. These factors drive tumor evolution through interactions between environmental selection and cellular adaptability. Consequently, the spatiotemporal heterogeneity undergoes changes [24,25,26]. In recent years, the TME has been recognized as an important participant in GBM pathogenesis and a potential therapeutic target [27]. Growing evidence suggests that differentiation among TME subsets within tumors may play a crucial role in treatment failure and recurrence [28,29,30]. The TME consists of several compartments containing intricate cellular and molecular interactions involving hypoxia, neovascularization, and energy metabolism. The upregulation of vascular endothelial growth factor (VEGF) by tumor cells in anoxic environments serves to induce angiogenesis. This leads to the formation of a locally chaotic microenvironment with high permeability and a disordered vascular structure, aimed at obtaining nutrients necessary to support tumor growth [31].

Currently, the majority of studies on gliomas’ internal heterogeneity or the TME primarily focus on fundamental research within the fields of oncology, immunology, and biology. These investigations often necessitate invasive and demanding procedures or biopsies for specimen acquisition, which are time-consuming and labor-intensive. Moreover, the limited sample size fails to adequately capture the intricate and dynamic spatial heterogeneity present throughout the entire tumor, ultimately compromising the accuracy of trial results and giving rise to significant ethical concerns. In recent years, positron emission computed tomography–magnetic resonance imaging (PET-MRI) has emerged with several compelling advantages, including enhanced soft tissue contrast and reduced exposure to ionizing radiation [32]. It enables effective initial disease characterization by providing a comprehensive multidimensional view of the brain structure, function, and dynamic activity [33]. However, high economic costs, as well as potential false-positive results in cases of inflammation, infection, or postoperative changes [34], impede the widespread adoption and routine use of PET-MRI. Therefore, advanced neuro-tumor imaging studies highlight the urgent requirement for advanced multi-parameter quantitative MRI (qMRI).

## 3. Glioma Invasiveness

The invasive pattern of glioma is characterized by diffuse infiltration along the perivascular space (PVS) and white matter tracts (WM), rather than focal invasion (Figure 1). However, the current imaging techniques lack precise demarcation beyond conventional enhanced MRI for the accurate determination of this boundary extension. Furthermore, the peritumbral edema surrounding high-grade gliomas (HGGs), particularly GBM, is composed of neoplastic infiltration and vasogenic edema [35,36]. Infiltrated tumor cells are present within the region of peritumbral edema, with some cells spreading along the PVS and WM. Notably, these malignant cells can extend their reach up to 3–5 cm beyond the glioma’s margin into the brain tissue via the WM or even invade the contralateral brain tissue through the corpus callosum. Consequently, assessing the tumor invasion extent accurately to achieve complete resection while minimizing nerve damage presents substantial challenges for clinicians.

The surgical objective is to achieve maximal tumor resection while ensuring safety. In HGGs, the current clinical strategy predominantly depends on conventional MRI-T1 weight-enhanced images to facilitate an “extended-scope resection”, which involves extending the surgical margin by approximately two centimeters beyond both the tumor mass and the surrounding edematous region. Recently, the application of sodium fluorescein (which exhibits yellow fluorescence) auxiliary technology has been utilized in conjunction with the surgical microscope to aid surgeons in accurately describing the tumor morphology, determining the precise tumor location and extent, and facilitating the complete resection of tumor tissue. For gliomas with indistinct boundaries or the involvement of critical brain functional areas, yellow fluorescence-assisted tumor resection can be performed, with a high degree of safety and reliability. Nevertheless, it is crucial not only to strictly control the timing of sodium fluorescein injection but also to discuss the optimal dosage for glioma patients. Moreover, operating under sodium fluorescein mode may carry the risk of damaging blood vessels, and its utility is limited in low-grade gliomas (LGGs) without enhancement. Therefore, there is an urgent need for advanced qMRI techniques that can provide a deeper understanding of the imaging and pathological characteristics associated with glioma invasion along WM, while enabling the more precise delineation of the resection scope.

## 4. Multi-Parameter qMRI in Glioma Microenvironment and Invasiveness

Over the past few decades, MRI has emerged as the predominant modality for medical imaging due to its exceptional soft tissue contrast and absence of ionizing radiation. Currently, MRI heavily relies on the subjective interpretation of signal features from various magnetic resonance sequences, including T1-weighted, T2-weighted, proton density-weighted, diffusion-weighted, and post-contrast/perfusion sequences. Radiologists typically utilize signals (such as high or low signals) to describe lesions and employ anticipated signals from different tissues and associated pathologies to make diagnoses and differential diagnoses. However, this subjective analysis based on relatively “weighted” images only captures a fraction of the comprehensive tissue information that can be provided by MRI techniques. Moreover, the current limitations in MRI techniques encompass variable image acquisition across scanners as well as acknowledged reader-to-reader variability in image interpretation. The field of qMRI presents a distinctive approach by providing a discrete analysis of tissue parameters, which holds great potential in addressing some of these limitations. Subsequently, we will explore the current research status of multi-parameter qMRI in evaluating the internal heterogeneity and invasiveness of gliomas, while also summarizing its distinctive features (Table 1).

### 4.1. Magnetic Resonance Fingerprinting (MRF)

**Figure 2:**
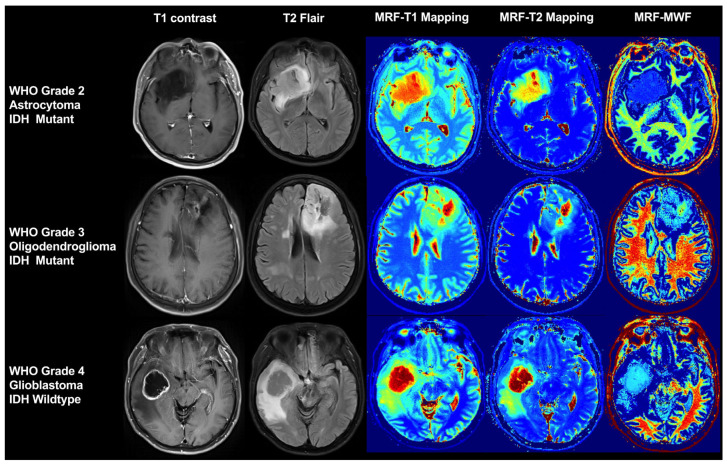
Representative cases utilizing MRF imaging across gliomas with different grades and IDH mutation statuses. Abbreviations: MRF, magnetic resonance fingerprinting. IDH, isocitrate dehydrogenase. WHO, World Health Organization. MWF, myelin water fraction.

The MRF [35,36,37,38] framework presents a novel approach for data acquisition, processing, visualization, and interpretation in the field of quantitative imaging. MRF has demonstrated high robustness, accuracy, and repeatability in both model and human validation studies. It leverages a single rapid sequence of pseudo-random and highly under-sampled samples to simultaneously capture the distinct temporal signal evolution from different tissues. The post-acquisition processing involves the application of a pattern matching algorithm, enabling the efficient and simultaneous measurement of multiple tissue attributes, such as relaxation, diffusion, perfusion, etc., thereby providing the comprehensive and quantitative characterization of the imaged tissue (Figure 2). This methodology ensures precise and reliable intrinsic histological characteristics. Since its introduction in 2013, MRF has been extensively employed in diverse clinical studies encompassing focal epilepsy [39,40,41], brain tumors [42,43], prostatic swelling [44], Parkinson’s disease [45,46,47], Alzheimer’s disease [48], psychiatric disorders [49], frontotemporal degeneration [50], and cerebral perfusion [51].

Numerous promising studies have been conducted on the practical application of MRF in brain tumors. Badve et al. [43] utilized steady-state free precession MRF sequences to scan patients with initial LGGs, HGGs, and brain metastases to obtain T1 mapping and T2 mapping. The results revealed that the mean T2 value effectively differentiated between LGG and metastatic tumors (172 ± 53 ms^−1^ vs. 105 ± 27 ms^−1^; *p* = 0.004), while the mean T1 value of the peri-tumor WM in LGG was significantly lower than that in GBM (1066 ± 218 ms^−1^ vs. 1578 ± 331 ms^−1^; *p* = 0.0004). Consistent findings were obtained in a similar study conducted by de Blank et al. [42], involving 23 children and adults, where it was observed that HGGs exhibited significantly higher T1 and T2 values compared to LGGs (T1: 1863 ± 70 ms^−1^ vs. 1355 ± 187 ms^−1^, *p* = 0.007; T2: 90 ± 13 ms^−1^ vs. 56 ± 19 ms^−1^, *p* = 0.013), and the peritumoral WM had a significantly lower T1 value in LGGs compared to HGGs (T1: 1154 ± 253 ms^−1^ vs. 1581 ± 476 ms^−1^, *p* = 0.039). A subsequent radiomics study based on the same data demonstrated that, when employing radiomic analysis, there was a significant improvement in the differential diagnosis efficiency among various tumor types, further highlighting the utility of MRF-based texture features in predicting survival rates among patients with GBM [52].

The study conducted by Haubold et al. [53] utilized multi-parametric MRI, MRF, and 18F-fluoroethy l-L-tyrosine (18F-FET) on a PET-MRI scanner, not only to assess the feasibility of molecular mutational states in 42 cases of primary gliomas but also to discriminate between HGG and LGG. Although the diagnostic efficacy in distinguishing HGG and LGG was moderate (AUC = 0.570–0.726), the evaluation of several crucial molecular mutation states revealed particularly significant findings. T1 and M0, including the MRF profiles, could be employed to predict 1p/19q mutations (AUC = 0.978), IDH1 mutations (AUC = 0.880), and ATRX mutations (AUC = 0.830). Similar outcomes were obtained by Tippareddy et al. [54], who investigated radiomic signatures derived from three-dimensional MRF T1 mapping to predict the IDH1 mutation status and correlate it with the overall survival rate. The study conducted by Marik et al. [55] involved the collection of MRF data from 17 glioma patients, which were then compared with positron emission tomography/computed tomography (PET-CT) in order to assess the predictive capability of region-defined MRF. These regions included the solid tumor region, peritumoral edema region, and contralateral normal brain parenchyma region. The results demonstrated accurate prediction exclusively based on T2 mapping for the solid tumor region, with a marginal enhancement observed when incorporating all MRF parameters collectively. This finding is consistent with previous studies [56,57] that suggest that T2 mapping is more suitable for tumor prediction, particularly in LGG, while T1 mapping is better suited for edema prediction.

Currently, T1 mapping and T2 mapping have been acquired using partial volume MRF data acquisition and a three-compartment model, which includes myelin (T1 = 130 ms, T2 = 20 ms), intracellular/extracellular water (cellular, T1 = 1300 ms, T2 = 130 ms), free water (T1 = 130 ms, T2 = 20 ms), and myelin water (T1 = 130 ms, T2 = 20 ms). Additionally, free water (T1 = 4500 ms, T2 = 500 ms) was incorporated to quantify the myelin water fraction (MWF), which has been utilized to assess WM development in children [37,58], evaluating WM maturation and integrity in typical development (TD) and leukodystrophies (LDs) [59,60], as well as multiple sclerosis (MS) [61]. The study conducted by Kim HG et al. [59] utilized histology to assess the MRF-derived MWF in mice, revealing significant correlations between the MWF and histologic measures of the myelin quantity, age, and the presence of leukodystrophy. These findings underscore the potential of the MRF-derived MWF as a rapid and non-invasive quantitative biomarker for the evaluation of the brain myelin content in both murine models and humans. Lancione M et al. [60] conducted a study on children with TD and LDs, demonstrating that the MWF based on MRF provides a WM maturation curve specific to the myelin sheath, which exhibits sensitivity to changes caused by LD and indicates its potential as a biomarker for WM disease. The study conducted by Lin Y et al. [61] revealed a significant difference in the MWF values between the normal-appearing white matter (NAWM) of MS patients and healthy controls, with respective values of 0.32 ± 0.025 and 0.25 ± 0.036. The mean MWF values in WM lesions were significantly smaller (0.034 ± 0.036) compared to those in NAWM. However, there is currently no existing literature on the application of the MWF in glioma WM infiltration, which highlights a direction for our future research efforts. Furthermore, Venugopal, K. et al. [62] proposed a blood vessel fingerprinting method based on dynamic susceptibility contrast (DSC) for the quantitative characterization of micro-vessels in gliomas. It quantifies major vascular biomarkers, rCBV, and the vessel radius in tumor and normal tissue, even when the tumor tissue has a leaky blood–brain barrier (BBB).

### 4.2. Diffusion-Weighted Imaging (DWI)

#### 4.2.1. Intra-Voxel Incoherent Motion (IVIM)

Vascularization plays a crucial role in the growth of infiltrating gliomas by transporting nutrients and removing metabolic waste from the tumor cells. The IVIM technique enables the quantification of perfusion-related microcirculation in blood capillaries at low b values (b < 200 s/mm^2^) and molecular water diffusion at high b values, without the need for contrast agents. Both parameters are closely associated with the microstructure and can be obtained within a short scan time [63,64,65]. Microvascular proliferation is a crucial histopathological feature when assessing glioma malignancy. The involvement of the VEGF signaling pathway in the process of neovascularization has been widely recognized [66]. The proangiogenic factor VEGF can induce a pathological enhancement in the microvascular permeability during tumor progression, leading to increased vascular leakage [67,68]. The hyperosmotic neovascularization primarily induces incoherent motion (rapid diffusion) at the capillary level of the glioma, thereby resulting in a disorganized pattern of movement. Furthermore, perfusion can also be observed as incoherent motion [69].

Numerous previous studies have confirmed the usefulness of IVIM in grading [64] and determining the IDH mutation statuses of gliomas [70,71]. Yu M et al. [71] integrated 2-hydroxyglutarate (2-HG) magnetic resonance spectroscopy (MRS) and IVIM and found an upward trend in the relative pseudo-diffusion coefficient (rD*) and the relative perfusion fraction value (rf) in IDH wildtype, which was significantly higher than that in IDH mutant. IDH wildtype gliomas exhibit a unique angiogenic gene expression signature, resulting in the enhanced migration of endothelial cells and remodeling of the matrix [72]. IDH mutations also enhance the accumulation of 2-HG, which is believed to trigger the degradation of hypoxia-inducible factor 1α (HIF-1α), potentially diminishing neovascularization [73]. However, the effect of 2-HG on HIF-1α is controversial [74], and Yu M et al.’s [71] study supports the theory that the accumulation of 2-HG may reduce HIF-1α. The study conducted by Sheng Y et al. [66] employed IVIM-derived perfusion and diffusion parameters to investigate their correlation with glioma histopathology. It was revealed that the rapid diffusion and perfusion fraction derived from IVIM can quantitatively reflect the proportion of VEGF-positive expressing cells (pVEGF) (r = 0.466, *p* = 0.007) and the percentage of MIB-1 (Ki67)-positive expression (pMIB-1) within the tumor. The findings imply that IVIM demonstrates superior diagnostic efficacy in assessing the malignancy of gliomas.

#### 4.2.2. Multiple Diffusion Model Imaging (DXI)

**Figure 3:**
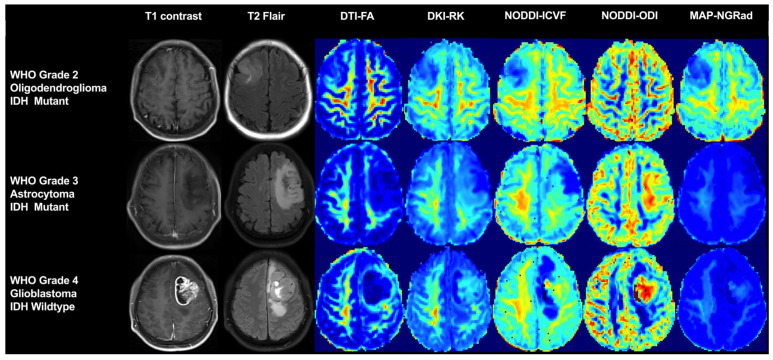
Representative cases utilizing multiple diffusion model imaging across gliomas with different grades and IDH mutation statuses. Abbreviations: IDH, isocitrate dehydrogenase. WHO, World Health Organization. DTI, diffusion tensor imaging. FA, fractional anisotropy. DKI, diffuse kurtosis imaging. RK, radial kurtosis. NODDI, neurite orientation dispersion and density imaging. ICVF, intracellular volume fraction. ODI, orientation dispersion index. MAP, mean apparent propagator. NGRad, radial non-Gaussianity.

Diffusion-weighted imaging (DWI) and diffusion tensor imaging (DTI) are the only techniques employed for the in vivo measurement of water molecule dispersion motion. Recently, DXI has emerged as a comprehensive approach that facilitates the acquisition of various diffusion models from a single scan (Figure 3), encompassing DTI, diffuse kurtosis imaging (DKI), mean apparent propagator (MAP) MRI, and neurite orientation dispersion and density imaging (NODDI). The aforementioned models have demonstrated exceptional performance in accurately predicting glioma genotyping [75] and effectively distinguishing GBM from isolated brain metastases [76].

The DTI sequence, which enables the application of more gradient fields in multiple directions compared to the DWI sequence, assumes a Gaussian dispersion distribution of water molecules within tumors, resulting in enhanced sensitivity towards the movement of water molecules. The application of DTI in CNS disorders yields exceptional imaging outcomes, particularly in discriminating between WM and gray matter, as well as tracing the trajectory of the WM. It facilitates an understanding of the compression displacement, infiltration, and destruction caused by lesions on WM. Moreover, it provides additional information for lesion diagnosis and differential diagnosis while serving as a basis for surgical planning and postoperative follow-up. The conventional DWI assumes a normal distribution of water molecule diffusion based on Gaussian motion. However, various factors, such as the cell ultrastructure, necrosis, and bleeding, lead to disordered non-Gaussian motion during the diffusion of water molecules [77]. Additionally, as a result of the anisotropic growth patterns observed in gliomas that infiltrate along the WM, tumor cells may demonstrate varying rates of invasion in different orientations.

DKI characterizes the degree to which diffusion deviates from the expected Gaussian distribution. DKI can be used to assess the fusion of complex tissue water by measuring kurtosis indicators, including the mean kurtosis (MK), radial kurtosis, and axial kurtosis. In recent years, DKI has demonstrated remarkable diagnostic accuracy in distinguishing between LGG and HGG, while effectively predicting their respective tumor grades [78,79]. The study conducted by Pang H et al. [79] revealed that DKI could effectively differentiate between LGG and HGG with IDH1 mutations based on their distinct nucleo-cytoplasmic ratios, rather than relying solely on cell counts. Specifically, MK parameters tend to exhibit higher values in HGG or gliomas with IDH wildtype compared to LGG or gliomas with IDH mutant, indicating an increase in microstructural complexity associated with malignancies, such as elevated cell proliferation, necrosis, and heterogeneous bleeding patterns, as well as cellular and myelin degradation products [80,81,82]. Additionally, DKI can serve as a valuable tool in evaluating WM damage and the myelin density [83].

MAP-MRI [84] is a novel computational framework based on Q-space data acquisition, which enables the evaluation of the non-Gaussian distribution of water molecules in brain tissue. It provides effective measurements of the spin displacement probability density function and quantifies essential indicators such as the mean azimuth shift, non-Gaussian property, q-space inverse variance (QIV), return to the origin probability (RTOP), etc. These indices reflect proton diffusion in complex microstructures, including diffusion confinement and multiple chambers. They have been utilized to detect changes in diffusion parameters caused by diffuse gliomas with varying grades or genetic characteristics [85,86]. Wang P et al. [85] conducted a study using MAP-MRI to identify the WHO grade of adult diffuse glioma and predict the IDH mutation status, as well as the 1p/19q co-deletion status. The results demonstrated that QIVmin or RTOPmax alone exhibited higher accuracy (AUC = 0.970) in predicting the IDH mutation status compared to the apparent diffusion coefficient (ADC) (DWI; AUC = 0.830) [87], mean diffusivity (MD) (DTI; AUC = 0.930) [88], and kurtosis anisotropy (KA) (NODDI; AUC = 0.76) [89]. Through comparison with the contralateral WM [84], it was speculated that this change reflected the diffuse invasion of the tumor and increased cell density in the extracellular matrix, which was somewhat associated with tumor heterogeneity. Avram AV et al. [90] discovered that a reduction in the RTOP indicated axon damage within the fiber bundle, accompanied by an increase in isotropic tissue.

NODDI is a practical diffusion MRI technique for the in vivo estimation of the microstructural complexity of dendrites and axons, particularly valuable in WM imaging. Its objective is to characterize various features of WM and enhance the characterization of WM. In comparison to DTI, NODDI utilizes different b-values to model brain tissue as three compartments exhibiting distinct diffusion properties [91]: the intracellular compartment modeled as restricted anisotropic non-Gaussian diffusion, the extracellular compartment modeled as hindered anisotropic Gaussian diffusion, and the cerebrospinal fluid compartment modeled as isotropic Gaussian diffusion. These compartments are derived from the neurite density index (NDI), orientation dispersion index (ODI), and isotropic volume fraction (ISOVF). The NDI reflects the axon density, the ODI indicates axon-oriented dispersion, while the ISOVF measures the extracellular components of the free water chamber [91]. This axon index directly correlates with the brain tissue microstructure [92] and serves as a more specific marker that may aid in identifying previously undetected lesions caused by glioma infiltration on MRI scans and improving our understanding of potential microstructure changes. Wen et al. [93] investigated the assessment of NODDI on the peritumoral infiltration of glioma using 7T-MRI and demonstrated a significant correlation between ISOVF maps and angiogenic edema. The invasion of high-signal tumor cells was observed along the WM within the extracellular volume map, intracellular volume, and signal reduction map, resulting in edema formation and neuronal loss. However, there is currently no existing quantitative characterization available for WM infiltration in glioma.

#### 4.2.3. Time-Dependent Diffusion-Weighted Imaging (TDS)

**Figure 4:**
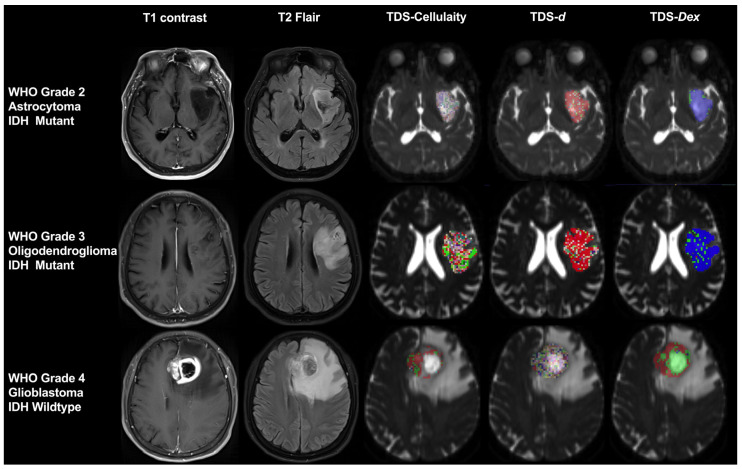
Representative cases utilizing TDS across gliomas with different grades and IDH mutation statuses. Abbreviations: TDS, time-dependent diffusion-weighted imaging. IDH, isocitrate dehydrogenase. WHO, World Health Organization. *d*, diameter. Dex, extracellular diffusion coefficient.

Recently, oscillating gradient spin echo (OGSE) diffusion sequences have been implemented in clinical MRI scanners. Pulsed gradient spin echo (PGSE) has been replaced with fast OGSE, which uses an extended diffusion pulse gradient from PGSE to achieve a shorter diffusion time. The combination of OGSE and PGSE has resulted in the development of a new diffusion MRI technique called TDS imaging. TDS allows for the collection of cellular information at different scales through organized arrangement and model fitting calculations based on discrete points collected over time (Figure 4) [94].

The internal structure of a lesion can be elucidated by employing the OGSE technique, which assesses alterations in the ADC values at varying diffusion times [95]. The ADC value is highly dependent on the effective diffusion time, which refers to the duration required for water molecules to diffuse within a living organism and explore the local environment. Recent research has demonstrated that OGSE-derived ADC values correlate with pathological indicators of microstructural characteristics in brain tumors [96,97], head and neck tumors [98], breast cancer [99], and prostate cancer [100]. While it is well documented that an increase in the tumor cell count corresponds to a decrease in the ADC, indicating an effective response to treatment when the ADC increases, the underlying pathophysiological mechanisms behind changes in the ADC values are not always fully understood. Various tissue properties, such as the cell density, cell size, nucleus size, membrane permeability, and necrosis, can influence the ADC values. The direct measurement of significant features related to the tissue microstructure at the cellular level can provide a more valuable assessment of tumor progression and the therapeutic response compared to non-specific ADC values. Xu et al. [94,100] validated the accuracy of “Imaging Microstructural Parameters Using Limited Spectrally Edited Diffusion” (IMPULSED) in depicting microstructural maps. The IMPULSED model is a two-compartment model that investigates how intracellular and extracellular water molecule diffusion affects the tissue microstructure. It allows for the calculation of various parameters, such as the cell density, diameter, intracellular volume fraction (Vin), and extracellular diffusion coefficient (Dex). This model has been successfully applied in studies on prostate cancer [100] and breast cancer [101]. However, there is currently no reported exploration of the intricate cellular microstructure found in adult GBM.

#### 4.2.4. Filtered-Exchange Imaging (FEXI)

The FEXI technique [102,103,104] was initially developed to assess water exchange across cellular membranes by leveraging diffusivity disparities between tissue compartments. In the context of the BBB, this enables the evaluation of cellular membranes and BBB separability. However, the modeling split lacks clear definition, and its accuracy, precision, and reproducibility have yet to be validated.

### 4.3. Resting-State Blood Oxygen Level-Dependent Functional Magnetic Resonance Imaging (r-BOLD-fMRI)

The NVC mechanism serves as the physiological basis for r-BOLD-fMRI [105]. Alterations in the ratio of oxygenated hemoglobin to deoxygenated hemoglobin result in uneven local magnetic susceptibility, ultimately leading to changes in signal intensity within the imaged brain region. Resting-state fMRI indirectly reflects the functional activation of brain regions through fluctuations in BOLD signals caused by variations in the hemoglobin concentration due to metabolism [106,107], which occur simultaneously across different brain regions. Studies conducted on mouse models [108] have demonstrated that glioma infiltration significantly disrupts neurovascular coupling and stimulus-induced hemodynamic responses. In human studies [109,110,111], r-BOLD-fMRI has revealed impaired vascular reactivity associated with gliomas, particularly affecting gray matter. Additionally, infiltrating glioma cells may release chemical mediators that directly modulate the astrocytic vascular reactivity or interfere with normal signaling molecule function [16,110,111,112,113,114]. Furthermore, a histological analysis of localized MRI biopsies [108] confirmed that vascular dysregulation is primarily caused by local tumor infiltration, rather than indirect mass effects or edema. The review conducted by De Simone M et al. [115] emphasized the potential of clustering fMRI time series in characterizing GBM and highlighted the ability of fMRI to provide insights into the functional characteristics of gliomas and their impacts on peritumoral brain activity. The observed changes in the BOLD signal may reflect alterations in local neuronal activity as well as vascular reactivity within and surrounding the tumor region. Although numerous structural disruptions in GBM are known to be accompanied by histopathological changes, the clinical relevance of quantifying and characterizing vascular dysfunction through BOLD functional imaging and assessing the severity of associated histopathological abnormalities related to tumor invasion remains uncertain.

### 4.4. MR Perfusion-Weighted Imaging (PWI)

There is increasing evidence [30,116] suggesting that the internal heterogeneity and differentiated development of glioma subsets play a crucial role in understanding treatment failure. The presence of specific histopathological features [117,118,119], such as pseudo-necrosis and micro-angiogenesis, contributes to the characteristic hypoxic and highly vascular nature of GBM, positioning it among the most oxygen-deprived and extensively perfused solid tumors. The poor prognosis of glioma, particularly GBM, can largely be attributed to its intra-tumoral heterogeneity. Apart from intrinsic molecular and genetic changes, glioma cells are also influenced by the TME, which primarily encompasses hypoxia, neovascularization generation, and energy metabolism conversion [120,121,122]. Stadlbauer A et al. [121] described three morphologically and functionally specialized tumor niches within GBM: perivascular, hypoxic, and vascular invasion niches. Perivascular niches are located in areas of tumor neovascularization, while anoxic niches exist within necrotic/anoxic regions of the tumor. In contrast, the aggressive vascular niche allows tumor cells to infiltrate normal blood vessels deeply into the brain parenchyma. Therefore, oxygen metabolism and neovascularization have potential as key biomarkers in elucidating the pathophysiological mechanisms underlying treatment resistance and relapse in this malignant refractory disease. However, due to their invasive nature (surgery or biopsy), limited reproducibility, and high cost (PET or PET-MRI), most available techniques are not well suited for non-invasive characterization within the human body. Consequently, utilizing multi-parameter MRI to obtain quantitative information about oxygen metabolism and neovascularization in gliomas has emerged as a prominent research focus area.

The most commonly employed MRI perfusion technique in clinical practice is DSC perfusion imaging [123]. It enables the assessment of hemodynamic parameters such as the cerebral blood volume (CBV) and CBF. Previous studies have demonstrated that DSC allows for the quantitative evaluation of tumor neovascularization and reflects the vascular supply status of tumor tissue. This has significant implications in assessing the glioma grade, prognosis, and distinguishing tumor recurrence from treatment-related changes [124,125]. Previously, we delineated the characteristics and mechanisms of glioma invasion, as well as investigating PVS invasion, using r-BOLD-fMRI. The study conducted by Petridis PD et al. [108] found no significant difference in regional CBV between the invasive hemisphere of glioma tumors and the healthy hemisphere among all enrolled patients with glioma. This investigation aimed to explore the feasibility of utilizing BOLD imaging to detect glioma-associated asynchrony in angio-dynamics, thereby distinguishing tumor tissue from healthy brain tissue. On the contrary, BOLD imaging clearly revealed vascular abnormalities beyond the conventional structural imaging anomalies. Stadlbauer, A’s team [121,122] combined these two techniques to characterize alterations in the internal microenvironment of GBM from the perspectives of oxygen metabolism and angiogenesis, respectively, presenting a novel concept for the comprehensive application of DSC technology. Dynamic contrast-enhanced (DCE) MRI [126] can quantitatively depict BBB damage, providing pharmacokinetic parameters such as the volume transfer constant (Ktrans), contrast agent reflux transfer constant (Kep), and extracellular volume fraction (Ve). Hillestad T et al. [127] employed the pixel-by-pixel fusion of the DCE parameters Ve and Ktrans to represent oxygen consumption and supply, respectively, developing an algorithm for the retrieval of surrogate measurements reflecting a continuous range of anoxia levels in patients with cervical cancer tumors. This approach was also utilized in Hompland et al.’s study [128], where it was integrated with multi-parameter MRI to demonstrate tumor hypoxia and aggressiveness in prostate cancer patients. Based on this premise, glioma perfusion imaging holds significant potential for clinical applications.

### 4.5. Chemical Exchange Saturation Transfer (CEST)

The extent of glioma resection is directly associated with patient survival, and the presence of residual tumor consistently results in early recurrence [129]. Therefore, the accurate delineation of tumor infiltration plays a crucial role in guiding clinical decision-making for glioma treatment. MRI is widely utilized in diagnosing glioma and has been applied in intraoperative neuro-navigation. However, as a result of the significant heterogeneity present within gliomas, certain regions within the same tumor may still maintain an intact BBB. This can lead to the enhanced signal on T1-weighted images being insufficiently precise in depicting tumor invasion, regardless of the grade of the glioma [130]. Furthermore, the distinction between tumor cell infiltration and peritumoral edema on T2-weighted images is not highly accurate. Amino acid PET scans utilize a radiolabeled tracer such as 18F-FET to visualize tumors based on their increased metabolic activity within the TME. The European Association for Neuro-Oncology (EANO) recommends this technique as the optimal method for the identification of glioma infiltration [131]. Nonetheless, limitations such as radiation exposure and high costs impede its widespread adoption and routine application [132].

CEST is a novel molecular MRI technology that offers distinct advantages over conventional MRI techniques, providing unique insights into metabolites such as free proteins, peptides, amides, glutamate, and creatine [133,134,135]. This enables the assessment of glioma metabolism at a biochemical level. Among various CEST imaging methods, amide proton transfer-weighted (APT) imaging plays a crucial role in grading, genotyping, differential diagnosis, and monitoring the prognosis and therapeutic response of glioma [136,137,138,139,140,141,142]. In a study by Yuan Y et al. [143], CEST combined with MRS was employed to predict glioma infiltration. The results showed significant correlations between the CEST (r = 0.736; *p* < 0.001), MRS (r = 0.495; *p* = 0.037), and 18F-FET findings. Moreover, the APT values were significantly higher in the HGG (3.923 ± 1.239) and IDH wildtype groups (3.932 ± 1.264) compared to the LGG (3.317 ± 0.868; *p* < 0.001) or IDH mutant groups (3.358 ± 0.847; *p* < 0.001). With its high diagnostic efficacy in predicting tumor invasion based on 18F-FET uptake, reaching 0.812 (95% CI: 0.808–0.815), CEST holds great promise as an advanced molecular imaging modality for the characterization of glioma invasion.

### 4.6. Multimodal Molecular Imaging

In recent years, chemical-, peptide-, antibody-, and nanoparticle-based probes have been designed to target specific molecules in gliomas and then visualized with multimodal molecular imaging techniques [144,145]. Functional nanoprobes [144] can not only enhance the permeability and retention of tumor microvessels but also target and localize tumors through specific binding to tumor-related biomarkers, such as tumor cell receptors, the tumor extracellular matrix, and enzymes. In addition, these contrast agents can be combined with therapeutic agents to mimic the spontaneous treatment of cancerous tissue. However, the limitations imposed by the BBB present significant challenges for the effective penetration of the currently developed contrast agents and their delivery to tumor sites in brain gliomas. This is also an important direction for future development.

**Table 1:** The characteristics of advanced multi-parametric quantitative MRI in glioma.

Biological Characteristic	MR Technique	Imaging Characteristics	References
Quantification	MRF	❿ Simultaneous measurement of multiple tissue properties: relaxation (T1 mapping, T2 mapping), diffusion (myelin water fraction), perfusion, etc. ❿ Absolute quantification	[35,36,37,38,39,40,41,42,43,44,45,46,47,48,49,50,51,52,53,54,55,56,57,58,59,60,61,62]
Diffusion	IVIM	❿ Vascular microstructural imaging, double exponential model ❿ Simultaneously detecting rapidly diffusing intravascular water molecules (perfusion) and slowly diffusing extravascular water molecules (diffusion) ❿ Without the utilization of contrast agents	[18,63,64,65,66,67,68,69,70,71,72,73,74]
	DTI	❿ Gaussian distribution, single exponential model ❿ Discrimination between WM and gray matter and tracing the trajectory of WM ❿ Quantifying the anisotropy of water molecule diffusion in multiple directions and evaluating tissue structure integrity ❿ Serving as a foundation for surgical planning and postoperative follow-up	[77]
	DKI	❿ Non-Gaussian distribution ❿ Reflecting the crossing of nerve fibers within the voxel (conventional DTI is unable to achieve this) ❿ Exploring microstructural complexity associated with malignancies, such as elevated cell proliferation, necrosis, and heterogeneous bleeding patterns ❿ Evaluating WM damage and myelin density	[78,79,80,81,82,83]
	MAP	❿ Non-Gaussian distribution, without making any assumptions about diffusion properties ❿ More precise and comprehensive parameters for microstructural organization ❿ Better understanding of the features related to the microstructure in WM	[84,85,86,87,88,89,90]
	NODDI	❿ Three-compartment model: intracellular compartment, extracellular compartment, and cerebrospinal fluid compartment ❿ Reflecting and discriminating the nerve density and the intersecting curvature of WM fiber tracts ❿ Estimation of the microstructural complexity of dendrites and axons	[91,92,93]
	TDS	❿ Combination of OGSE and PGSE (conventional DWI) ❿ Reflecting the real situation of water molecular motion in the tissue microstructure ❿ Overcoming the limitation whereby conventional DWI can only measure the ADC value, which reflects the comprehensive effect of water molecular diffusion in the tissue structure in the single dimension of the diffusion time ❿ The IMPULSED model is the most clinically used and mature model to calculate the cell density, d, Vin, and Dex	[94,95,96,97,98,99,100,101]
	FEXI	❿ Evaluation of cellular membrane and BBB permeability ❿ No clear modeling division	[102,103,104]
Blood oxygen	*r*-BOLD-fMRI	❿ Providing insights into blood oxygen level of tumor and peritumoral edema ❿ Revealing impaired cerebral vascular reactivity associated with gliomas	[16,105,106,107,108,109,110,111,112,113,114,115,121,122]
Perfusion	DSC	❿ Based on the assumption of an intact BBB ❿ Assessment of hemodynamic parameters, such as CBV and CBF ❿ Quantitative evaluation of tumor neovascularization and reflecting the vascular supply status of tumor tissue ❿ Requiring the injection of contrast agents	[121,122,123,124,125]
	DCE	❿ Quantitatively depicting BBB damage ❿ Assessment of pharmacokinetic parameters, such as Ktrans and Ve ❿ Quantitative evaluation of tumor neovascularization and reflecting the degree of permeability of tumor tissue ❿ Requiring the injection of contrast agents	[126,127,128]
Metabolism	CEST	❿ Assessment of glioma metabolism at a biochemical level ❿ Providing unique insights into metabolites such as free proteins, peptides, amides, glutamate, and creatine	[17,129,130,131,132,133,134,135,136,137,138,139,140,141,142,143]

Abbreviations: MRI, magnetic resonance imaging; MRF, magnetic resonance fingerprinting; IVIM, intra-voxel incoherent motion; DTI, diffusion tensor imaging; WM, white matter; DKI, diffusion tensor imaging; MAP, mean apparent propagator; NODDI, neurite orientation dispersion and density imaging; DWI, diffusion-weighted imaging; TDS, time-dependent diffusion-weighted imaging; OGSE, oscillating gradient spin echo; PGSE, pulsed gradient spin echo; ADC, apparent diffusion coefficient; d, diameter; Vin, intracellular volume fraction; Dex, extracellular diffusion coefficient; FEXI, filtered-exchange imaging; BBB, blood–brain barrier; DSC, dynamic susceptibility contrast; CBV, cerebral blood volume; CBF, cerebral blood flow; DCE, dynamic contrast-enhanced; Ktrans, volume transfer constant; Ve, extracellular volume fraction; r-BOLD-fMRI, resting-state blood oxygen level-dependent functional magnetic resonance imaging; CEST, chemical exchange saturation transfer.

## 5. Conclusions

In conclusion, non-invasive and advanced multi-parametric qMRI, including MRF, DWI, PWI, BOLD, CEST, etc., along with molecular imaging techniques such as nanoprobes, not only effectively address the limitations of subjective assessment in traditional imaging but also accurately analyze changes and differences in microstructures like cells and microvessels within the tumor and its periphery. This is achieved from a multidimensional biological perspective, encompassing diffusion, perfusion, and metabolism, which is more consistent with histopathology and even molecular biology. Thus, multi-parametric qMRI plays a crucial role in the visual assessment of glioma malignancy prior to surgery, in assessing the extent of resection after surgery, and in determining the efficacy of radiotherapy and chemotherapy. In the future, it is imperative to prioritize the integration of multimodal qMRI techniques with advanced tools such as habitat and digital pathology for a comprehensive study of the intrinsic heterogeneity and invasive properties of gliomas. Furthermore, there is a need to enhance the delivery of contrast agents, including molecular probes, in brain gliomas to enable precise imaging and efficient treatment. 

## Data Availability

Not applicable.

## References

[1] Sabeghi P., Zarand P., Zargham S., Golestany B., Shariat A., Chang M., Yang E., Rajagopalan P., Phung D.C., Gholamrezanezhad A. (2024). Advances in Neuro-Oncological Imaging: An Update on Diagnostic Approach to Brain Tumors. Cancers.

[2] Zinnhardt B., Roncaroli F., Foray C., Agushi E., Osrah B., Hugon G., Jacobs A.H., Winkeler A. (2021). Imaging of the glioma microenvironment by TSPO PET. Eur. J. Nucl. Med. Mol. Imaging.

[3] Ostrom Q.T., Truitt G., Gittleman H., Brat D.J., Kruchko C., Wilson R., Barnholtz-Sloan J.S. (2020). Relative survival after diagnosis with a primary brain or other central nervous system tumor in the National Program of Cancer Registries, 2004 to 2014. Neuro-Oncol. Pract..

[4] Ostrom Q.T., Patil N., Cioffi G., Waite K., Kruchko C., Barnholtz-Sloan J.S. (2020). CBTRUS Statistical Report: Primary Brain and Other Central Nervous System Tumors Diagnosed in the United States in 2013–2017. Neuro-Oncol..

[5] Gurrieri L., Mercatali L., Ibrahim T., Fausti V., Dall’agata M., Riva N., Ranallo N., Pasini G., Tazzari M., Foca F. (2023). Immuno markers in newly diagnosed glioblastoma patients underwent Stupp protocol after neurosurgery: A retrospective series. J. Neuro-Oncol..

[6] Jiang T., Nam D., Ram Z., Poon W.-S., Wang J., Boldbaatar D., Mao Y., Ma W., Mao Q., You Y. (2021). Clinical practice guidelines for the management of adult diffuse gliomas. Cancer Lett..

[7] Tan A.C., Ashley D.M., López G.Y., Malinzak M., Friedman H.S., Khasraw M. (2020). Management of glioblastoma: State of the art and future directions. CA A Cancer J. Clin..

[8] Louis D.N., Perry A., Wesseling P., Brat D.J., Cree I.A., Figarella-Branger D., Hawkins C., Ng H.K., Pfister S.M., Reifenberger G. (2021). The 2021 WHO Classification of Tumors of the Central Nervous System: A summary. Neuro-Oncol..

[9] Louis D.N., Perry A., Reifenberger G., Von Deimling A., Figarella-Branger D., Cavenee W.K., Ohgaki H., Wiestler O.D., Kleihues P., Ellison D.W. (2016). The 2016 World Health Organization Classification of Tumors of the Central Nervous System: A summary. Acta Neuropathol..

[10] De Simone M., Conti V., Palermo G., De Maria L., Iaconetta G. (2023). Advancements in Glioma Care: Focus on Emerging Neurosurgical Techniques. Biomedicines.

[11] Gao M., Lin Y., Liu X., Zhao Z., Zhu Z., Zhang H., Ban Y., Bie Y., He X., Sun X. (2021). TERT Mutation Is Accompanied by Neutrophil Infiltration and Contributes to Poor Survival in Isocitrate Dehydrogenase Wild-Type Glioma. Front. Cell Dev. Biol..

[12] Tang F., Chen X., Liu J.-S., Liu Z.-Y., Yang J.-Z., Wang Z.-F., Li Z.-Q. (2023). TERT mutations-associated alterations in clinical characteristics, immune environment and therapy response in glioblastomas. Discov. Oncol..

[13] Yamashita K., Hatae R., Kikuchi K., Kuga D., Hata N., Yamamoto H., Obara M., Yoshimoto K., Ishigami K., Togao O. (2023). Predicting TERT promoter mutation status using 1H-MR spectroscopy and stretched-exponential model of diffusion-weighted imaging in IDH-wildtype diffuse astrocytic glioma without intense enhancement. Neuroradiology.

[14] Johnson D.R., Giannini C., Vaubel R.A., Morris J.M., Eckel L.J., Kaufmann T.J., Guerin J.B. (2022). A Radiologist’s Guide to the 2021 WHO Central Nervous System Tumor Classification: Part I—Key Concepts and the Spectrum of Diffuse Gliomas. Radiology.

[15] Minami J.K., Morrow D., Bayley N.A., Fernandez E.G., Salinas J.J., Tse C., Zhu H., Su B., Plawat R., Jones A. (2023). CDKN2A deletion remodels lipid metabolism to prime glioblastoma for ferroptosis. Cancer Cell.

[16] Englander Z.K., Horenstein C.I., Bowden S.G., Chow D.S., Otten M.L., Lignelli A., Bruce J.N., Canoll P., Grinband J. (2018). Extent of BOLD Vascular Dysregulation Is Greater in Diffuse Gliomas without Isocitrate Dehydrogenase 1 R132H Mutation. Radiology.

[17] Mancini L., Casagranda S., Gautier G., Peter P., Lopez B., Thorne L., McEvoy A., Miserocchi A., Samandouras G., Kitchen N. (2022). CEST MRI provides amide/amine surrogate biomarkers for treatment-naïve glioma sub-typing. Eur. J. Nucl. Med. Mol. Imaging.

[18] Guo D., Jiang B. (2023). Noninvasively evaluating the grade and IDH mutation status of gliomas by using mono-exponential, bi-exponential diffusion-weighted imaging and three-dimensional pseudo-continuous arterial spin labeling. Eur. J. Radiol..

[19] Nicholson J.G., Fine H.A. (2021). Diffuse Glioma Heterogeneity and Its Therapeutic Implications. Cancer Discov..

[20] Clarke R. (2017). Introduction: Cancer Gene Networks. Methods Mol. Biol..

[21] Dagogo-Jack I., Shaw A.T. (2017). Tumour heterogeneity and resistance to cancer therapies. Nat. Rev. Clin. Oncol..

[22] Prasetyanti P.R., Medema J.P. (2017). Intra-tumor heterogeneity from a cancer stem cell perspective. Mol. Cancer.

[23] Chaligne R., Gaiti F., Silverbush D., Schiffman J.S., Weisman H.R., Kluegel L., Gritsch S., Deochand S.D., Castro L.N.G., Richman A.R. (2021). Epigenetic encoding, heritability and plasticity of glioma transcriptional cell states. Nat. Genet..

[24] Gatenby R.A., Grove O., Gillies R.J. (2013). Quantitative imaging in cancer evolution and ecology. Radiology.

[25] Kim J.Y., Gatenby R.A. (2017). Quantitative Clinical Imaging Methods for Monitoring Intratumoral Evolution. Methods Mol. Biol..

[26] Vitale I., Shema E., Loi S., Galluzzi L. (2021). Intratumoral heterogeneity in cancer progression and response to immunotherapy. Nat. Med..

[27] Bikfalvi A., da Costa C.A., Avril T., Barnier J.V., Bauchet L., Brisson L., Cartron P.F., Castel H., Chevet E., Chneiweiss H. (2023). Challenges in glioblastoma research: Focus on the tumor microenvironment. Trends Cancer.

[28] Goenka A., Tiek D., Song X., Huang T., Hu B., Cheng S.-Y. (2021). The Many Facets of Therapy Resistance and Tumor Recurrence in Glioblastoma. Cells.

[29] Valtorta S., Salvatore D., Rainone P., Belloli S., Bertoli G., Moresco R.M. (2020). Molecular and Cellular Complexity of Glioma. Focus on Tumour Microenvironment and the Use of Molecular and Imaging Biomarkers to Overcome Treatment Resistance. Int. J. Mol. Sci..

[30] Hardee M.E., Zagzag D. (2012). Mechanisms of glioma-associated neovascularization. Am. J. Pathol..

[31] Perrin S.L., Samuel M.S., Koszyca B., Brown M.P., Ebert L.M., Oksdath M., Gomez G.A. (2019). Glioblastoma heterogeneity and the tumour microenvironment: Implications for preclinical research and development of new treatments. Biochem. Soc. Trans..

[32] Yuan Y. (2016). Spatial Heterogeneity in the Tumor Microenvironment. Cold Spring Harb. Perspect. Med..

[33] Sabeghi P., Katal S., Chen M., Taravat F., Werner T.J., Saboury B., Gholamrezanezhad A., Alavi A. (2023). Update on Positron Emission Tomography/Magnetic Resonance Imaging: Cancer and Inflammation Imaging in the Clinic. Magn. Reson. Imaging Clin. North Am..

[34] Miller-Thomas M.M., Benzinger T.L.S. (2017). Neurologic Applications of PET/MR Imaging. Magn. Reson. Imaging Clin. N. Am..

[35] Ma D., Gulani V., Seiberlich N., Liu K., Sunshine J.L., Duerk J.L., Griswold M.A. (2013). Magnetic resonance fingerprinting. Nature.

[36] Tippareddy C., Zhao W., Sunshine J.L., Griswold M., Ma D., Badve C. (2021). Magnetic resonance fingerprinting: An overview. Eur. J. Nucl. Med. Mol. Imaging.

[37] Wang C.Y., Coppo S., Mehta B.B., Seiberlich N., Yu X., Griswold M.A. (2019). Magnetic resonance fingerprinting with quadratic RF phase for measurement of T_2_* simultaneously with δ_f_, T_1_, and T_2_. Magn. Reson. Med..

[38] Springer E., Cardoso P.L., Strasser B., Bogner W., Preusser M., Widhalm G., Nittka M., Koerzdoerfer G., Szomolanyi P., Hangel G. (2022). MR Fingerprinting-A Radiogenomic Marker for Diffuse Gliomas. Cancers.

[39] Liao C., Wang K., Cao X., Li Y., Wu D., Ye H., Ding Q., He H., Zhong J. (2018). Detection of Lesions in Mesial Temporal Lobe Epilepsy by Using MR Fingerprinting. Radiology.

[40] Adler S., Lorio S., Jacques T.S., Benova B., Gunny R., Cross J.H., Baldeweg T., Carmichael D.W. (2017). Towards in vivo focal cortical dysplasia phenotyping using quantitative MRI. Neuroimage. Clin..

[41] Ma D., Jones S.E., Deshmane A., Sakaie K., Pierre E.Y., Larvie M., McGivney D., Blümcke I., Krishnan B., Lowe M. (2019). Development of high-resolution 3D MR fingerprinting for detection and characterization of epileptic lesions. J. Magn. Reson. Imaging.

[42] de Blank P., Badve C., Gold D.R., Stearns D., Sunshine J., Dastmalchian S., Tomei K., Sloan A.E., Barnholtz-Sloan J.S., Lane A. (2019). Magnetic Resonance Fingerprinting to Characterize Childhood and Young Adult Brain Tumors. Pediatr. Neurosurg..

[43] Badve C., Yu A., Dastmalchian S., Rogers M., Ma D., Jiang Y., Margevicius S., Pahwa S., Lu Z., Schluchter M. (2017). MR Fingerprinting of Adult Brain Tumors: Initial Experience. Am. J. Neuroradiol..

[44] Yu A.C., Badve C., Ponsky L.E., Pahwa S., Dastmalchian S., Rogers M., Jiang Y., Margevicius S., Schluchter M., Tabayoyong W. (2017). Development of a Combined MR Fingerprinting and Diffusion Examination for Prostate Cancer. Radiology.

[45] Keil V.C., Bakoeva S.P., Jurcoane A., Doneva M., Amthor T., Koken P., Mädler B., Lüchters G., Block W., Wüllner U. (2020). A pilot study of magnetic resonance fingerprinting in Parkinson’s disease. NMR Biomed..

[46] Lee J.E., Cho K.H., Song S.K., Kim H.J., Lee H.S., Sohn Y.H., Lee P.H. (2013). Exploratory analysis of neuropsychological and neuroanatomical correlates of progressive mild cognitive impairment in Parkinson’s disease. J. Neurol. Neurosurg. Psychiatry.

[47] Chung S.J., Yoo H.S., Lee Y.H., Lee H.S., Ye B.S., Sohn Y.H., Kwon H., Lee P.H. (2019). Frontal atrophy as a marker for dementia conversion in Parkinson’s disease with mild cognitive impairment. Hum. Brain Mapp..

[48] Möller C., Vrenken H., Jiskoot L., Versteeg A., Barkhof F., Scheltens P., van der Flier W.M. (2013). Different patterns of gray matter atrophy in early- and late-onset Alzheimer’s disease. Neurobiol. Aging.

[49] Sasabayashi D., Takahashi T., Takayanagi Y., Suzuki M. (2021). Anomalous brain gyrification patterns in major psychiatric disorders: A systematic review and transdiagnostic integration. Transl. Psychiatry.

[50] Keil V.C., Bakoeva S.P., Jurcoane A., Doneva M., Amthor T., Koken P., Mädler B., Block W., Fimmers R., Fliessbach K. (2019). MR fingerprinting as a diagnostic tool in patients with frontotemporal lobe degeneration: A pilot study. Nmr Biomed..

[51] Su P., Mao D., Liu P., Li Y., Pinho M.C., Welch B.G., Lu H. (2017). Multiparametric estimation of brain hemodynamics with MR fingerprinting ASL. Magn. Reson. Med..

[52] Dastmalchian S., Kilinc O., Onyewadume L., Tippareddy C., McGivney D., Ma D., Griswold M., Sunshine J., Gulani V., Barnholtz-Sloan J.S. (2021). Radiomic analysis of magnetic resonance fingerprinting in adult brain tumors. Eur. J. Nucl. Med. Mol. Imaging.

[53] Haubold J., Demircioglu A., Gratz M., Glas M., Wrede K., Sure U., Antoch G., Keyvani K., Nittka M., Kannengiesser S. (2020). Non-invasive tumor decoding and phenotyping of cerebral gliomas utilizing multiparametric 18F-FET PET-MRI and MR Fingerprinting. Eur. J. Nucl. Med. Mol. Imaging.

[54] Tippareddy C., Onyewadume L., Sloan A.E., Wang G.-M., Patil N.T., Hu S., Barnholtz-Sloan J.S., Boyacıoğlu R., Gulani V., Sunshine J. (2023). Novel 3D magnetic resonance fingerprinting radiomics in adult brain tumors: A feasibility study. Eur. Radiol..

[55] Marik W., Cardoso P.L., Springer E., Bogner W., Preusser M., Widhalm G., Hangel G., Hainfellner J.A., Rausch I., Weber M. (2023). Evaluation of Gliomas with Magnetic Resonance Fingerprinting with PET Correlation—A Comparative Study. Cancers.

[56] Ding H., Velasco C., Ye H., Lindner T., Grech-Sollars M., O’callaghan J., Hiley C., Chouhan M.D., Niendorf T., Koh D.-M. (2021). Current Applications and Future Development of Magnetic Resonance Fingerprinting in Diagnosis, Characterization, and Response Monitoring in Cancer. Cancers.

[57] Kern M., Auer T.A., Picht T., Misch M., Wiener E. (2020). T2 mapping of molecular subtypes of WHO grade II/III gliomas. BMC Neurol..

[58] Chen Y., Chen M.-H., Baluyot K.R., Potts T.M., Jimenez J., Lin W. (2019). MR fingerprinting enables quantitative measures of brain tissue relaxation times and myelin water fraction in the first five years of life. Neuroimage.

[59] Kim H.G., Han D., Kim J., Choi J.-S., Cho K.-O. (2023). 3D MR fingerprinting-derived myelin water fraction characterizing brain development and leukodystrophy. J. Transl. Med..

[60] Lancione M., Cencini M., Scaffei E., Cipriano E., Buonincontri G., Schulte R.F., Pirkl C.M., Buchignani B., Pasquariello R., Canapicchi R. (2024). Magnetic resonance fingerprinting-based myelin water fraction mapping for the assessment of white matter maturation and integrity in typical development and leukodystrophies. NMR Biomed..

[61] Lin Y., Chan K.H., Mak H.K., Yau K.X., Cao P. (2024). Quantitative myelin water assessment for multiple sclerosis using multi-inversion magnetic resonance fingerprinting. Med. Phys..

[62] Venugopal K., Arzanforoosh F., van Dorth D., Smits M., van Osch M.J.P., Hernandez-Tamames J.A., Warnert E.A.H., Poot D.H.J. (2023). MR Vascular Fingerprinting with Hybrid Gradient–Spin Echo Dynamic Susceptibility Contrast MRI for Characterization of Microvasculature in Gliomas. Cancers.

[63] Nilsson M., Englund E., Szczepankiewicz F., van Westen D., Sundgren P.C. (2018). Imaging brain tumour microstructure. Neuroimage.

[64] Federau C., Meuli R., O’Brien K., Maeder P., Hagmann P. (2014). Perfusion measurement in brain gliomas with intravoxel incoherent motion MRI. Am. J. Neuroradiol..

[65] Le Bihan D. (2019). What can we see with IVIM MRI?. Neuroimage.

[66] Sheng Y., Dang X., Zhang H., Rui W., Wang J., Cheng H., Qiu T., Zhang Y., Ding Y., Yao Z. (2023). Correlations between intravoxel incoherent motion–derived fast diffusion and perfusion fraction parameters and VEGF- and MIB-1-positive rates in brain gliomas: An intraoperative MR-navigated, biopsy-based histopathologic study. Eur. Radiol..

[67] Hectors S.J., Gordic S., Semaan S., Bane O., Hirten R., Jia X., Colombel J.-F., Taouli B. (2019). Diffusion and perfusion MRI quantification in ileal Crohn’s disease. Eur. Radiol..

[68] Siveen K.S., Prabhu K., Krishnankutty R., Kuttikrishnan S., Tsakou M., Alali F.Q., Dermime S., Mohammad R.M., Uddin S. (2017). Vascular endothelial growth factor (VEGF) signaling in tumour vascularization: Potential and challenges. Curr. Vasc. Pharmacol..

[69] Bisdas S., Braun C., Skardelly M., Schittenhelm J., Teo T.H., Thng C.H., Klose U., Koh T.S. (2014). Correlative assessment of tumor microcirculation using contrast-enhanced perfusion MRI and intravoxel incoherent motion diffusion-weighted MRI: Is there a link between them?. NMR Biomed..

[70] Lu J., Li X., Li H. (2021). Perfusion parameters derived from MRI for preoperative prediction of IDH mutation and MGMT promoter methylation status in glioblastomas. Magn. Reson. Imaging.

[71] Yu M., Ge Y., Wang Z., Zhang Y., Hou X., Chen H., Chen X., Ji N., Li X., Shen H. (2024). The diagnostic efficiency of integration of 2HG MRS and IVIM versus individual parameters for predicting IDH mutation status in gliomas in clinical scenarios: A retrospective study. J. Neuro-Oncol..

[72] van Santwijk L., Kouwenberg V., Meijer F., Smits M., Henssen D. (2022). A systematic review and meta-analysis on the differentiation of glioma grade and mutational status by use of perfusion-based magnetic resonance imaging. Insights Imaging.

[73] Koivunen P., Lee S., Duncan C.G., Lopez G., Lu G., Ramkissoon S., Losman J.A., Joensuu P., Bergmann U., Gross S. (2012). Transformation by the (R)-enantiomer of 2-hydroxyglutarate linked to EGLN activation. Nature.

[74] Ježek P. (2020). 2-hydroxyglutarate in cancer cells. Antioxid. Redox Signal.

[75] Gao A., Zhang H., Yan X., Wang S., Chen Q., Gao E., Qi J., Bai J., Zhang Y., Cheng J. (2022). Whole-Tumor Histogram Analysis of Multiple Diffusion Metrics for Glioma Genotyping. Radiology.

[76] Qi J., Wang P., Zhao G., Gao E., Zhao K., Gao A., Bai J., Zhang H., Yang G., Zhang Y. (2023). Histogram Analysis Based on Neurite Orientation Dispersion and Density MR Imaging for Differentiation Between Glioblastoma Multiforme and Solitary Brain Metastasis and Comparison of the Diagnostic Performance of Two ROI Placements. J. Magn. Reson. Imaging.

[77] Zhang J., Chen X., Chen D., Wang Z., Li S., Zhu W. (2018). Grading and proliferation assessment of diffuse astrocytic tumors with monoexponential, biexponential, and stretched-exponential diffusion-weighted imaging and diffusion kurtosis imaging. Eur. J. Radiol..

[78] Falk Delgado A., Nilsson M., van Westen D., Falk Delgado A. (2018). Glioma Grade Discrimination with MR Diffusion Kurtosis Imaging: A Meta-Analysis of Diagnostic Accuracy. Radiology.

[79] Pang H., Dang X., Ren Y., Yao Z., Shen Y., Feng X., Wang Z. (2023). DKI can distinguish high-grade gliomas from IDH1-mutant low-grade gliomas and correlate with their different nuclear-to-cytoplasm ratio: A localized biopsy-based study. Eur. Radiol..

[80] Hempel J.-M., Bisdas S., Schittenhelm J., Brendle C., Bender B., Wassmann H., Skardelly M., Tabatabai G., Vega S.C., Ernemann U. (2017). In vivo molecular profiling of human glioma using diffusion kurtosis imaging. J. Neuro-Oncol..

[81] Bai Y., Lin Y., Tian J., Shi D., Cheng J., Haacke E.M., Hong X., Ma B., Zhou J., Wang M. (2016). Grading of Gliomas by Using Monoexponential, Biexponential, and Stretched Exponential Diffusion-weighted MR Imaging and Diffusion Kurtosis MR Imaging. Radiology.

[82] Kleihues P., Soylemezoglu F., Schäuble B., Scheithauer B.W., Burger P.C. (1995). Histopathology, classification, and grading of gliomas. Glia.

[83] Lesbats C., Kelly C.L., Czanner G., Poptani H. (2020). Diffusion kurtosis imaging for characterizing tumor heterogeneity in an intracranial rat glioblastoma model. Nmr Biomed..

[84] Wang P., He J., Ma X., Weng L., Wu Q., Zhao P., Ban C., Hao X., Hao Z., Yuan P. (2022). Applying MAP-MRI to Identify the WHO Grade and Main Genetic Features of Adult-type Diffuse Gliomas: A Comparison of Three Diffusion-weighted MRI Models. Acad. Radiol..

[85] Wang P., Weng L., Xie S., He J., Ma X., Li B., Yuan P., Wang S., Zhang H., Niu G. (2021). Primary application of mean apparent propagator-MRI diffusion model in the grading of diffuse glioma. Eur. J. Radiol..

[86] Sun Y., Su C., Deng K., Hu X., Xue Y., Jiang R. (2022). Mean apparent propagator-MRI in evaluation of glioma grade, cellular proliferation, and IDH-1 gene mutation status. Eur. Radiol..

[87] Maynard J., Okuchi S., Wastling S., Al Busaidi A., Almossawi O., Mbatha W., Brandner S., Jaunmuktane Z., Koc A.M., Mancini L. (2020). World Health Organization Grade II/III Glioma Molecular Status: Prediction by MRI Morphologic Features and Apparent Diffusion Coefficient. Radiology.

[88] Tan W.L., Huang W.Y., Yin B., Xiong J., Wu J.S., Geng D.Y. (2014). Can diffusion tensor imaging noninvasively detect IDH1 gene mutations in astrogliomas? A retrospective study of 112 cases. Am. J. Neuroradiol..

[89] Figini M., Riva M., Graham M., Castelli G.M., Fernandes B., Grimaldi M., Baselli G., Pessina F., Bello L., Zhang H. (2018). Prediction of Isocitrate Dehydrogenase Genotype in Brain Gliomas with MRI: Single-Shell versus Multishell Diffusion Models. Radiology.

[90] Avram A.V., Sarlls J.E., Barnett A.S., Özarslan E., Thomas C., Irfanoglu M.O., Hutchinson E., Pierpaoli C., Basser P.J. (2016). Clinical feasibility of using mean apparent propagator (MAP) MRI to characterize brain tissue microstructure. Neuroimage.

[91] Zhang H., Schneider T., Wheeler-Kingshott C.A., Alexander D.C. (2012). NODDI: Practical in vivo neurite orientation dispersion and density imaging of the human brain. Neuroimage.

[92] Andica C., Kamagata K., Kirino E., Uchida W., Irie R., Murata S., Aoki S. (2021). Neurite orientation dispersion and density imaging reveals white matter microstructural alterations in adults with autism. Mol. Autism.

[93] Wen Q., Kelley D.A.C., Banerjee S., Lupo J.M., Chang S.M., Xu D., Hess C.P., Nelson S.J. (2015). Clinically feasible NODDI characterization of glioma using multiband EPI at 7 T. NeuroImage Clin..

[94] Jiang X., Li H., Xie J., Zhao P., Gore J.C., Xu J. (2016). Quantification of cell size using temporal diffusion spectroscopy. Magn. Reson. Med..

[95] Ejima F., Fukukura Y., Kamimura K., Nakajo M., Ayukawa T., Kanzaki F., Yanazume S., Kobayashi H., Kitazono I., Imai H. (2024). Oscillating Gradient Diffusion-Weighted MRI for Risk Stratification of Uterine Endometrial Cancer. J. Magn. Reson. Imaging.

[96] Maekawa T., Hori M., Murata K., Feiweier T., Kamiya K., Andica C., Hagiwara A., Fujita S., Koshino S., Akashi T. (2020). Differentiation of high-grade and low-grade intra-axial brain tumors by time-dependent diffusion MRI. Magn. Reson. Imaging.

[97] Kamimura K., Kamimura Y., Nakano T., Hasegawa T., Nakajo M., Yamada C., Akune K., Ejima F., Ayukawa T., Ito S. (2023). Differentiating brain metastasis from glioblastoma by time-dependent diffusion MRI. Cancer Imaging.

[98] Iima M., Yamamoto A., Kataoka M., Yamada Y., Omori K., Feiweier T., Togashi K. (2018). Time-dependent diffusion MRI to distinguish malignant from benign head and neck tumors. J. Magn. Reson. Imaging.

[99] Teruel J.R., Cho G.Y., Moccaldi RT M., Goa P.E., Bathen T.F., Feiweier T., Kim S.G., Moy L., Sigmund E.E. (2017). Stimulated echo diffusion tensor imaging (STEAM-DTI) with varying diffusion times as a probe of breast tissue. J. Magn. Reson. Imaging.

[100] Wu D., Jiang K., Li H., Zhang Z., Ba R., Zhang Y., Hsu Y.-C., Sun Y., Zhang Y.-D. (2022). Time-Dependent Diffusion MRI for Quantitative Microstructural Mapping of Prostate Cancer. Radiology.

[101] Ba R., Wang X., Zhang Z., Li Q., Sun Y., Zhang J., Wu D. (2023). Diffusion-time dependent diffusion MRI: Effect of diffusion-time on microstructural mapping and prediction of prognostic features in breast cancer. Eur. Radiol..

[102] Lasič S., Nilsson M., Lätt J., Ståhlberg F., Topgaard D. (2011). Apparent exchange rate mapping with diffusion MRI. Magn. Reson. Med..

[103] Nilsson M., Lätt J., van Westen D., Brockstedt S., Lasič S., Ståhlberg F., Topgaard D. (2013). Noninvasive mapping of water diffusional exchange in the human brain using filter-exchange imaging. Magn. Reson. Med..

[104] Powell E., Ohene Y., Battiston M., Dickie B.R., Parkes L.M., Parker G.J.M. (2023). Blood-brain barrier water exchange measurements using FEXI: Impact of modeling paradigm and relaxation time effects. Magn. Reson. Med..

[105] Howarth C., Mishra A., Hall C.N. (2021). More than just summed neuronal activity: How multiple cell types shape the BOLD response. Philos. Trans. R. Soc. Lond. B Biol. Sci..

[106] Kim S., Ogawa S. (2012). Biophysical and physiological origins of blood oxygenation level-dependent fMRI signals. J. Cereb. Blood Flow. Metab..

[107] Jian Z., Wang X., Tian M., Liu Y., Yao H., Gong L., Li B. (2022). Review of the Research Progress of Human Brain Oxygen Extraction Fraction by Magnetic Resonance Imaging. Oxid. Med. Cell Longev..

[108] Petridis P.D., Horenstein C.I., Pereira B., Wu P.B., Samanamud J., Marie T., Boyett D., Sudhakar T.D., A Sheth S., McKhann G.M. (2022). BOLD asynchrony elucidates tumor burden in IDH-mutated gliomas. Neuro-Oncol..

[109] Montgomery M.K., Kim S.H., Dovas A., Zhao H.T., Goldberg A.R., Xu W., Yagielski A.J., Cambareri M.K., Patel K.B., Mela A. (2020). Glioma-Induced Alterations in Neuronal Activity and Neurovascular Coupling during Disease Progression. Cell Rep..

[110] Agarwal S., Sair H.I., Pillai J.J. (2017). The Resting-State Functional Magnetic Resonance Imaging Regional Homogeneity Metrics—Kendall’s Coefficient of Concordance-Regional Homogeneity and Coherence-Regional Homogeneity—Are Valid Indicators of Tumor-Related Neurovascular Uncoupling. Brain Connect..

[111] Agarwal S., Lu H., Pillai J.J. (2017). Value of Frequency Domain Resting-State Functional Magnetic Resonance Imaging Metrics Amplitude of Low-Frequency Fluctuation and Fractional Amplitude of Low-Frequency Fluctuation in the Assessment of Brain Tumor-Induced Neurovascular Uncoupling. Brain Connect..

[112] Pillai J.J., Zacà D. (2012). Comparison of BOLD Cerebrovascular Reactivity Mapping and DSC MR Perfusion Imaging for Prediction of Neurovascular Uncoupling Potential in Brain Tumors. Technol. Cancer Res. Treat..

[113] Zacà D., Jovicich J., Nadar S.R., Voyvodic J.T., Pillai J.J. (2014). Cerebrovascular reactivity mapping in patients with low grade gliomas undergoing presurgical sensorimotor mapping with BOLD fMRI. J. Magn. Reson. Imaging.

[114] Iranmahboob A., Peck K.K., Brennan N.P., Karimi S., Fisicaro R., Hou B., Holodny A.I. (2016). Vascular Reactivity Maps in Patients with Gliomas Using Breath-Holding BOLD fMRI. J. Neuroimaging Off. J. Am. Soc. Neuroimaging.

[115] De Simone M., Iaconetta G., Palermo G., Fiorindi A., Schaller K., De Maria L. (2024). Clustering Functional Magnetic Resonance Imaging Time Series in Glioblastoma Characterization: A Review of the Evolution, Applications, and Potentials. Brain Sci..

[116] Stupp R., Mason W.P., van den Bent M.J., Weller M., Fisher B., Taphoorn M.J.B., Belanger K., Brandes A.A., Marosi C., Bogdahn U. (2005). Radiotherapy plus concomitant and adjuvant temozolomide for glioblastoma. N. Engl. J. Med..

[117] Greaves M., Maley C.C. (2012). Clonal evolution in cancer. Nature.

[118] Gillies R.J., Verduzco D., Gatenby R.A. (2012). Evolutionary dynamics of carcinogenesis and why targeted therapy does not work. Nat. Rev. Cancer.

[119] Marusyk A., Almendro V., Polyak K. (2012). Intra-tumour heterogeneity: A looking glass for cancer?. Nat. Rev. Cancer.

[120] Quail D.F., Joyce J.A. (2017). The Microenvironmental Landscape of Brain Tumors. Cancer Cell.

[121] Stadlbauer A., Zimmermann M., Doerfler A., Oberndorfer S., Buchfelder M., Coras R., Kitzwögerer M., Roessler K. (2018). Intratumoral heterogeneity of oxygen metabolism and neovascularization uncovers 2 survival-relevant subgroups of IDH1 wild-type glioblastoma. Neuro-Oncol..

[122] Stadlbauer A., Oberndorfer S., Zimmermann M., Renner B., Buchfelder M., Heinz G., Doerfler A., Kleindienst A., Roessler K. (2020). Physiologic MR imaging of the tumor microenvironment revealed switching of metabolic phenotype upon recurrence of glioblastoma in humans. J. Cereb. Blood Flow Metab..

[123] Soliman R.K., Gamal S.A., Essa A.-H.A., Othman M.H. (2018). Preoperative Grading of Glioma Using Dynamic Susceptibility Contrast MRI: Relative Cerebral Blood Volume Analysis of Intra-tumoural and Peri-tumoural Tissue. Clin. Neurol. Neurosurg..

[124] Yuan Y., Zeng D., Liu Y., Tao J., Zhang Y., Yang J., Lkhagvadorj T., Yin Z., Wang S. (2020). DWI and IVIM are predictors of Ki67 proliferation index: Direct comparison of MRI images and pathological slices in a murine model of rhabdomyosarcoma. Eur. Radiol..

[125] Bai Y., Lin Y., Zhang W., Kong L., Wang L., Zuo P., Vallines I., Schmitt B., Tian J., Song X. (2017). Noninvasive amide proton transfer magnetic resonance imaging in evaluating the grading and cellularity of gliomas. Oncotarget.

[126] Cha S., Johnson G., Wadghiri Y.Z., Jin O., Babb J., Zagzag D., Turnbull D.H. (2003). Dynamic, contrast-enhanced perfusion MRI in mouse gliomas: Correlation with histopathology. Magn. Reson. Med..

[127] Hillestad T., Hompland T., Fjeldbo C.S., Skingen V.E., Salberg U.B., Aarnes E.-K., Nilsen A., Lund K.V., Evensen T.S., Kristensen G.B. (2020). MRI Distinguishes Tumor Hypoxia Levels of Different Prognostic and Biological Significance in Cervical Cancer. Cancer Res..

[128] Hompland T., Hole K.H., Ragnum H.B., Aarnes E.-K., Vlatkovic L., Lie A.K., Patzke S., Brennhovd B., Seierstad T., Lyng H. (2018). Combined MR Imaging of Oxygen Consumption and Supply Reveals Tumor Hypoxia and Aggressiveness in Prostate Cancer Patients. Cancer Res..

[129] Molinaro A.M., Hervey-Jumper S., Morshed R.A., Young J., Han S.J., Chunduru P., Zhang Y., Phillips J.J., Shai A., Lafontaine M. (2020). Association of Maximal Extent of Resection of Contrast-Enhanced and Non–Contrast-Enhanced Tumor with Survival Within Molecular Subgroups of Patients with Newly Diagnosed Glioblastoma. JAMA Oncol..

[130] Gao X., Yue Q., Liu Y., Fan D., Fan K., Li S., Qian J., Han L., Fang F., Xu F. (2018). Image-guided chemotherapy with specifically tuned blood brain barrier permeability in glioma margins. Theranostics.

[131] Albert N.L., Weller M., Suchorska B., Galldiks N., Soffietti R., Kim M.M., la Fougère C., Pope W., Law I., Arbizu J. (2016). Response Assessment in Neuro-Oncology working group and European Association for Neuro-Oncology recommendations for the clinical use of PET imaging in gliomas. Neuro-Oncol..

[132] Schiff D., Van den Bent M., Vogelbaum M.A., Wick W., Miller C.R., Taphoorn M., Pope W., Brown P.D., Platten M., Jalali R. (2019). Recent developments and future directions in adult lower-grade gliomas: Society for Neuro-Oncology (SNO) and European Association of Neuro-Oncology (EANO) consensus. Neuro-Oncol..

[133] Cai K., Haris M., Singh A., Kogan F., Greenberg J.H., Hariharan H., A Detre J., Reddy R. (2012). Magnetic resonance imaging of glutamate. Nat. Med..

[134] Heo H., Lee D., Zhang Y., Zhao X., Jiang S., Chen M., Zhou J. (2017). Insight into the quantitative metrics of chemical exchange saturation transfer (CEST) imaging. Magn. Reson. Med..

[135] Cai K., Singh A., Poptani H., Li W., Yang S., Lu Y., Hariharan H., Zhou X.J., Reddy R. (2015). CEST signal at 2 ppm (CEST@2ppm) from Z-spectral fitting correlates with creatine distribution in brain tumor. Nmr Biomed..

[136] Wu M., Jiang T., Guo M., Duan Y., Zhuo Z., Weng J., Xie C., Sun J., Li J., Cheng D. (2024). Amide proton transfer-weighted imaging and derived radiomics in the classification of adult-type diffuse gliomas. Eur. Radiol..

[137] Hou H., Chen W., Diao Y., Wang Y., Zhang L., Wang L., Xu M., Yu J., Song T., Liu Y. (2023). 3D Amide Proton Transfer-Weighted Imaging for Grading Glioma and Correlating IDH Mutation Status: Added Value to 3D Pseudocontinuous Arterial Spin Labelling Perfusion. Mol. Imaging Biol..

[138] Guo H., Liu J., Hu J., Zhang H., Zhao W., Gao M., Zhang Y., Yang G., Cui Y. (2022). Diagnostic performance of gliomas grading and IDH status decoding A comparison between 3D amide proton transfer APT and four diffusion-weighted MRI models. J. Magn. Reson. Imaging.

[139] Xu Z., Ke C., Liu J., Xu S., Han L., Yang Y., Qian L., Liu X., Zheng H., Lv X. (2020). Diagnostic performance between MR amide proton transfer (APT) and diffusion kurtosis imaging (DKI) in glioma grading and IDH mutation status prediction at 3 T. Eur. J. Radiol..

[140] Krijnen W.P. (2006). Some Results on Mean Square Error for Factor Score Prediction. Psychometrika.

[141] Sakata A., Okada T., Yamamoto A., Kanagaki M., Fushimi Y., Okada T., Dodo T., Arakawa Y., Schmitt B., Miyamoto S. (2015). Grading glial tumors with amide proton transfer MR imaging: Different analytical approaches. J. Neuro-Oncol..

[142] Paech D., Windschuh J., Oberhollenzer J., Dreher C., Sahm F., Meissner J.-E., Goerke S., Schuenke P., Zaiss M., Regnery S. (2018). Assessing the predictability of IDH mutation and MGMT methylation status in glioma patients using relaxation-compensated multipool CEST MRI at 7.0 T. Neuro-Oncol..

[143] Yuan Y., Yu Y., Guo Y., Chu Y., Chang J., Hsu Y., Liebig P.A., Xiong J., Yu W., Feng D. (2022). Noninvasive Delineation of Glioma Infiltration with Combined 7T Chemical Exchange Saturation Transfer Imaging and MR Spectroscopy: A Diagnostic Accuracy Study. Metabolites.

[144] Tang T., Chang B., Zhang M., Sun T. (2021). Nanoprobe-mediated precise imaging and therapy of glioma. Nanoscale Horiz..

[145] Li J., Huang S., Shao K., Liu Y., An S., Kuang Y., Guo Y., Ma H., Wang X., Jiang C. (2013). A choline derivate-modified nanoprobe for glioma diagnosis using MRI. Sci. Rep..

